# *Ranunculus ternatus* Thunb extract attenuates renal fibrosis of diabetic nephropathy via inhibiting SMYD2

**DOI:** 10.1080/13880209.2022.2030759

**Published:** 2022-02-10

**Authors:** Weiwei Xu, Rui Peng, Siyu Chen, Congcong Wu, Xiaoxiao Wang, Ting Yu, Jiuying Jian, Ni Zhang, Siyang Zuo, Min Chen, Bing Guo, Lirong Liu

**Affiliations:** a Center for Clinical Laboratories, The Affiliated Hospital of Guizhou Medical University, Guiyang, China; b Department of Clinical Hematology, School of Clinical Laboratory Science, Guizhou Medical University, Guiyang, China; c Department of Pathophysiology, Guizhou Medical University, Guiyang, China; d Laboratory of Pathogenesis Research, Drug Prevention and Treatment of Major Diseases, Guizhou Medical University, Guiyang, China

**Keywords:** RTT, anti-fibrosis, anti-inflammatory, lysine methyltransferases

## Abstract

**Context:**

*Ranunculus ternatus* Thunb (Ranunculaceae), (RTT) is used clinically for the treatment of tuberculosis or as tumour adjuvant therapy, but its potential effect on diabetic nephropathy (DN) has not been studied.

**Objective:**

To investigate the effect of RTT extract in renal fibrosis of DN.

**Materials and methods:**

C57BL/6 mice were randomly divided into four groups (*n* = 12). Diabetes mellitus (DM) mice were induced by streptozotocin (STZ, 55 mg/kg/day) for five consecutive days and treated by RTT extract (2 g/kg). Afterward, blood glucose, HE and Masson staining were assayed. The expression levels of Vimentin, ɑ-SMA, TNF-ɑ, NF-κB p-p65, NF-κB p65, SMYD2, H3K36me3, H3K4me3 were determined by western blots. Firbronectin was respectively assayed by western blot and immunofluorescent staining.

**Results:**

RTT extract significantly ameliorated renal injury and renal fibrosis in the renal tissue of STZ-induced diabetic mice as demonstrated by the decreased expression level of Fibronectin (65%), Vimentin and α-SMA (75% & 53%). In addition, the levels of TNF-α (57%), NF-κB p-p65 and NF-κB p65 (35% & 25%) were elevated in the DN mice. Importantly, these were alleviated after RTT extract treatment. Moreover, we observed that the protein levels of SMYD2 (30%), H3K36me3 and H3K4me3 (53% & 75%) were reduced in DN mice after treatment with RTT extract.

**Discussion and conclusions:**

RTT extract mediates antifibrotic effects and anti-inflammatory responses in STZ-induced DN mainly through suppressing SMYD2 activation and H3K36me3 and H3K4me3 protein expression. RTT extract might have therapeutic potential against high glucose-induced nephropathy.

## Introduction

Diabetes mellitus (DM) is a metabolic disease syndrome caused by a combination of hereditary and environmental factors. According to the International Diabetes Federation (IDF), the number of DM patients worldwide hit 451 million in 2017, and it is expected to reach 693 million by 2045 (Cho et al. 2018). Diabetic nephropathy (DN), a common microvascular complication in DM patients, is the leading cause of end-stage renal disease (ESRD) (Zhang et al. 2016). During the progression of DN, renal injury is strongly associated with renal fibrosis, especially tubulointerstitial fibrosis (Zeisberg and Neilson 2010). Renal fibrosis is characterized by excessive deposition of extracellular matrix (ECM), along with increased numbers of activated myofibroblasts in kidney parenchyma (Humphreys 2018). Multiple studies have implicated that myofibroblasts are the main effector cells that synthesize and secrete ECM (Sun et al. 2016). *In vivo*, fibroblasts will proliferate and activate transdifferentiated myofibroblasts, under the stimulation of high glucose, high fat or other conditions. Additional epithelial-mesenchymal transdifferentiation of renal tubular epithelial cells also can produce myofibroblasts. Both of them could produce and release a variety of growth factors and inflammatory cytokines. The activation and proliferation of renal interstitial fibroblasts can be induced by symptomatic factors and vasoactive substances (Lovisa et al. 2015). Although several signalling mediators (such as transforming growth factor-β1/Smad3, nuclear factor κ-light-chain-enhancer of activated B cells [NF-κB], signal transduction and transcriptional activators 3) are involved in driving renal fibrosis; the detailed molecular mechanism underlying renal fibrosis in DM has not been fully determined.

NF-κB expresses various genes encoding proteins, playing a vital role in processes of immunity, inflammation, cell survival and apoptosis (Chen et al. 2012). Dysregulation of NF-κB has been implicated in the mechanisms involved in inflammation under certain pathological conditions such as renal fibrosis or ischaemic injury (Li et al. 2019). The activation of NF-κB by various cellular stress stimuli, like the release of cytokines (tumour necrosis factor-α, TNF-α; interleukin-1, 6, IL-1, 6), can mediate transcription of some genes, which would increase the cellular stress responses (Li et al. 2019). For example, TNF-α can rapidly activate NF-κB in the cytoplasm during the development of DN, exert the transcriptional effect role of NF-κB on numerous cytokines, promote inflammatory cell infiltration, and induce renal fibrosis (Verhelst et al. 2011). Therefore, NF-κB signalling pathways play an important role in regulating the occurrence and development of renal fibrosis. More interestingly, NF-κB signalling pathway can be regulated by lysine methyltransferases (LMTs) (Lewis et al. 2008; Lu and Stark 2015). Lysine methyltransferase 2 (SMYD2), a kind of LMTs, contains a SET domain, which can methylate histones (H3K36 and H3K4) and non-histone proteins (transcription factors NF-κB and p53, etc.). Moreover, SMYD2 is up-regulated in the renal epithelial cells of autosomal dominant polycystic kidney mice and can delay the growth of renal cysts, and it is activated by methylating the NF-κB p65 subunit to promote the proliferation and survival of cystic renal epithelial cells (Malik et al. 2017). In our previous work, we have found that the expression of SMYD2 was significantly increased in the kidney tissue of diabetic mice induced by STZ (Jian et al. 2020). This suggests that histone methylation performs a crucial role in promoting fibroblast activation and ECM synthesis, and the occurrence and development of renal fibrosis may be related to the activation of SMYD2.

Rosiglitazone and Metformin are currently the most effective treatments used for progressive DN. However, synthetic drugs have multiple side effects in the treatment of DN, such as cardiovascular diseases or gastrointestinal adverse effects (Kores et al. 2021; Tarry-Adkins et al. 2021). Therefore, there is an urgent need to explore novel agents or adjuvants for DN. *Ranunculus ternatus* Thunb (Ranunculaceae) (RTT), is widely distributed in Guizhou, Yunnan, Henan and other provinces of China. Thus, it is cheap and easy to obtain. Clinically, RTT extract has a variety of pharmacological effects, such as clearing heat and detoxification, detumescence, relieving cough and expectorant. Therefore, it has mainly been used for the treatment of tuberculosis or as adjuvant treatment of tumour (Niu et al. 2013). In addition, it is reported that RTT extract can inhibit the growth and colony formation of various types of cancer cells and induce apoptosis (Hao et al. 2017), and regulate immune function (Lv et al. 2010). Phytochemical studies have confirmed that RTT extract contains polysaccharides, flavonoids, glycosides, organic acid, ester, amino acids, constant and trace elements (Fang et al. 2020). Polysaccharides widely exist in the natural plants of the natural world, which are especially one of the active ingredients in multiple Chinese medicines. They are characterized by having various biological activities, such as antitumor, anti-inflammatory, hypoglycaemic and hypolipidemic activities (Lee et al. 2010; Mima 2013). Hyperglycaemic control in diabetes is the key to preventing the occurrence and development of DN. It is reported that the inflammation induced by hyperglycaemia and dyslipidemia metabolism may play a significant role in the development of vascular complications including DN (Mima 2013).

To date, the potential effects of RTT extract in preventing diabetes-induced renal fibrosis has not been established. In this study, we aim to explore the effect of RTT extract on renal function and histopathological changes of renal tissue in STZ-induced diabetic mice and investigate the regulatory role of RTT extract in preventing renal fibrosis of DN and its possible mechanism.

## Materials and methods

### Materials

RTT was purchased from the pharmacy department of the Affiliated Hospital of Guizhou Medical University (Guizhou, China). Streptozotocin (STZ) was purchased from Sigma (Cat. No. 206075, St. Louis, MO, USA). Blood glucose (BG) test kit was purchased from Johnson (Cat. No. 95035, Milpitas, CA, USA). HE (Cat. No. G1120) and Masson (Cat. No. G1340) staining kits were purchased from Solarbio (Beijing, China). Immunohistochemistry (IHC) (Cat. No. SB-0023) detection reagents and DAB colour reagents (Cat. No. D10294) were purchased from ZSGB-BIO (Beijing, China). Antibodies against Fibronectin (FN) (Cat. No. ab2413), Vimentin (Cat. No. V5255), TNF-α (Cat. No. ab1793), H3K36me3 (Cat. No. ab9050) and H3K4me3 (Cat. No. ab8580) were purchased from Abcam (Cambridge, MA, USA). Antibodies against α-smooth muscle actin (α-SMA) was from Sigma (Cat. No. D4K9N, St. Louis, MO, USA), NF-κB p-p65 (Cat. No. S536/93H1), NF-κB p65 (Cat. No. D14E12) and SMYD2 (Cat. No. D14H7) were from Cell Signalling Technology (Danvers, MA, USA), and β-actin was from BIOSS (Cat. No. BS-0061R, Beijing, China).

### Preparation of RTT extract

**Figure 1.**
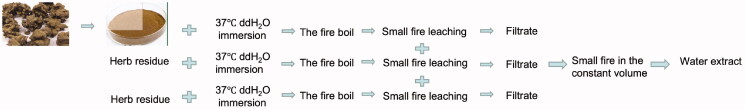
Preparation method of RTT extract.

RTT was purchased from the pharmacy of Affiliated Hospital of Guizhou Medical University (Guizhou, China). The RTT extract was prepared by the standard procedure (Figure 1). In brief, the dried root tubers (100 g) of the RTT extract were ground into coarse particles and then extracted three-times with distilled water under reflux. The combined supernatant extracts were evaporated at 50–60 °C to give a concentrated aqueous extract.

### Animals and treatment

**Figure 2.**
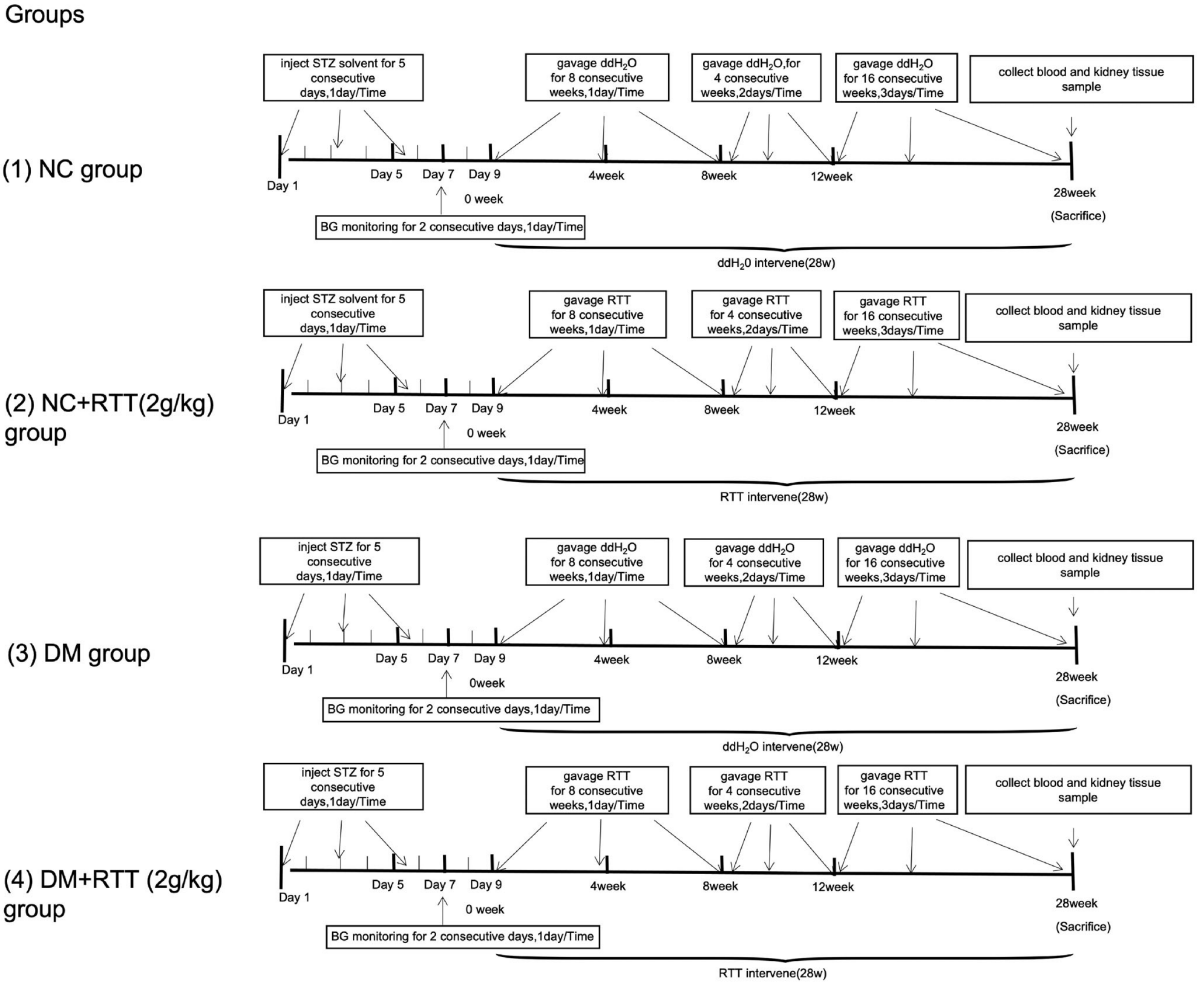
Experimental scheme of RTT extract treatment to examine potential for the improvement of renal injury in STZ-induced diabetic mice.

Forty-eight healthy and specific pathogen-free (SPF) male C57BL/6 mice weighing 16–20 g were provided by SBF Biotechnology Co. Ltd (Beijing, China). All animals were kept in a sanitary environment at a constant temperature of 20–25 °C in the Animal Centre of Guizhou Medical University (Guizhou, China). All animal studies followed the rules set out by China’s National Health and Medical Research Council’s Code for the Care and Use of Animals for Scientific Purpose: SYXK (QIAN) 2018-0001. All mice were randomly divided into normal control group (NC), RTT extract control group (NC + RTT), diabetes mellitus group (DM) and RTT extract treatment group (DM + RTT), 12 mice in each group. DM mice were induced by injecting STZ through the tail vein at a dose of 55 mg/kg/day, while NC mice were injected with an equal volume of solvent. Fasting blood glucose (BG) was measured after 48 h, and those with BG greater than or equal to 16.7 mmol/L and positive urine glucose was positive were used for experiments. STZ-induced diabetic mice and normal mice were treated by gavage with RTT extract (2 g/kg) at different times (1–8 weeks, once a day; 9–12 weeks, once every 2 days; 13–28 weeks, once every 3 days). The NC and DM groups were given the same amount of distilled water by gavage (Figure 2). At 28 weeks, all animals were killed, kidneys were taken for histologic evaluation and protein analysis, and blood was collected for BG.

### Biochemical assays

Blood and urine glucose was measured by the oxidase method with a 1650 automatic clinical chemistry analyzer (Beckman Instruments, CA, USA).

### Histochemical and immunofluorescent staining

Renal tissues were dehydrated and embedded in paraffin after being fixed in 4% neutral formaldehyde. After continuous sectioning, 4 μm sections were unfolded and fixed on the glass sheet. HE and Masson staining were performed according to the manufacturer’s instructions (Solarbio, Beijing, China). Morphological and structural changes of renal tissue were examined by light microscopy. Immunofluorescent staining was carried out following the reported in our previous work (Pang et al. 2009). Rabbit anti-Fibronectin antibody was used for immunofluorescence staining. All images were captured using an optical microscope (Olympus BX61, Olympus, Japan).

### Western blot analysis

Renal tissues were homogenised using lysis buffer, sonicated, and centrifuged at 12,000 *g* for 20 min at 4 °C. Protein concentration was quantified by bovine serum albumin, then measured by absorption in a spectrophotometer. After that, all samples were collected and frozen at −20 °C for subsequent use. Proteins were assessed by standard western blot as described (Li et al. 2015). Briefly, the protein was separated by SDS-PAGE and transferred to polyvinylidene difluoride membranes (PVDF, Millipore, Bedford, MA, USA). Membranes were incubated with 5% non-fat milk for 1 h at room temperature, then incubated with primary antibodies against Fibronectin (1:5000), Vimentin (1:1000), TNF-ɑ (1:1000), ɑ-SMA (1:750), NF-κB p-p65 (1:1000), NF-κB p65 (1:1000), SMYD2 (1:1000), H3K36me3 (1:1000), H3K4me3 (1:1000) and β-actin (1:1000) overnight at 4 °C, followed by secondary antibodies conjugated with horseradish peroxidase for 1 h at room temperature. Bound antibodies were detected using chemiluminescence (Millipore, USA), then processed by grey scanning with ImageJ (National Institutes of Health, USA). All the target proteins were normalized by β-actin and calculated as the percentage of the control.

### Statistical analysis

All data were presented as means ± standard deviation (SD) obtained from at least three independent experiments. Statistical analysis was performed using Student’s *t*-test (GraphPad Software, Inc.). *p*-Values of <0.05 were considered statistically significant in this study.

## Results

### RTT extract treatment reduced renal injury in STZ-induced diabetic mice

**Figure 3.**
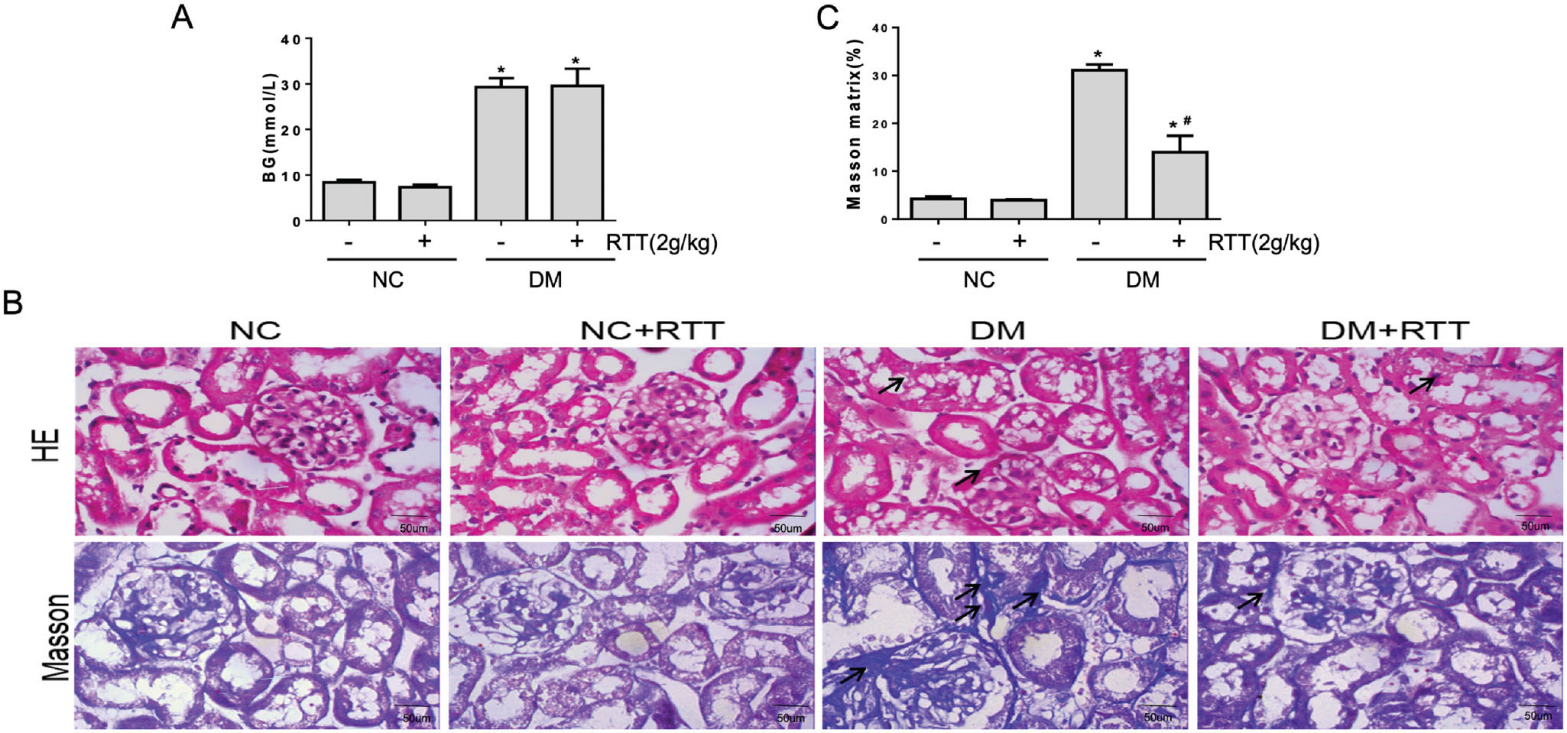
RTT extract treatment improved renal injury in STZ-induced diabetic mice. (A) Blood glucose (BG) from STZ-induced diabetic mice after 28 weeks of RTT extract (2 g/kg) treatment (*n* = 12, means ± SD, *p < 0.05 vs. NC group); (B) Representative photomicrographs (original magnification, 400×) of HE and Masson staining (blue) of kidney sections in STZ-induced diabetic mice after 28 weeks of RTT extract (2 g/kg) treatment (*n* = 12). (C) Quantification of Masson staining (*n* = 12, *p < 0.05 vs. NC group; #*p* < 0.05 vs. DM group).

RTT extract treatment had no effect on the increased BG levels in STZ-induced diabetic mice (Figure 3(A)). HE staining showed STZ-induced diabetic mice had disordered renal tubular structure, irregular thickening of the basement membrane, disorder of renal tubular epithelial cells, vacuolar and granular degeneration, and infiltration of inflammatory cells in tubulointerstitium. RTT extract treatment alleviated the irregular thickening of the renal tubular basement membrane, reduced the vacuole and granular degeneration of renal tubular epithelial cells as well as the infiltration of renal tubular interstitial inflammatory cells. Masson staining showed that there were many vacuoles and granular degeneration in renal tubular epithelial cells of STZ induced diabetic mice, and evidence of renal interstitial fibrosis was also observed. However, renal fibrosis was significantly relieved after RTT extract treatment (Figure 3(B,C)).

### RTT extract inhibited renal fibrosis in STZ-induced diabetic mice

**Figure 4.**
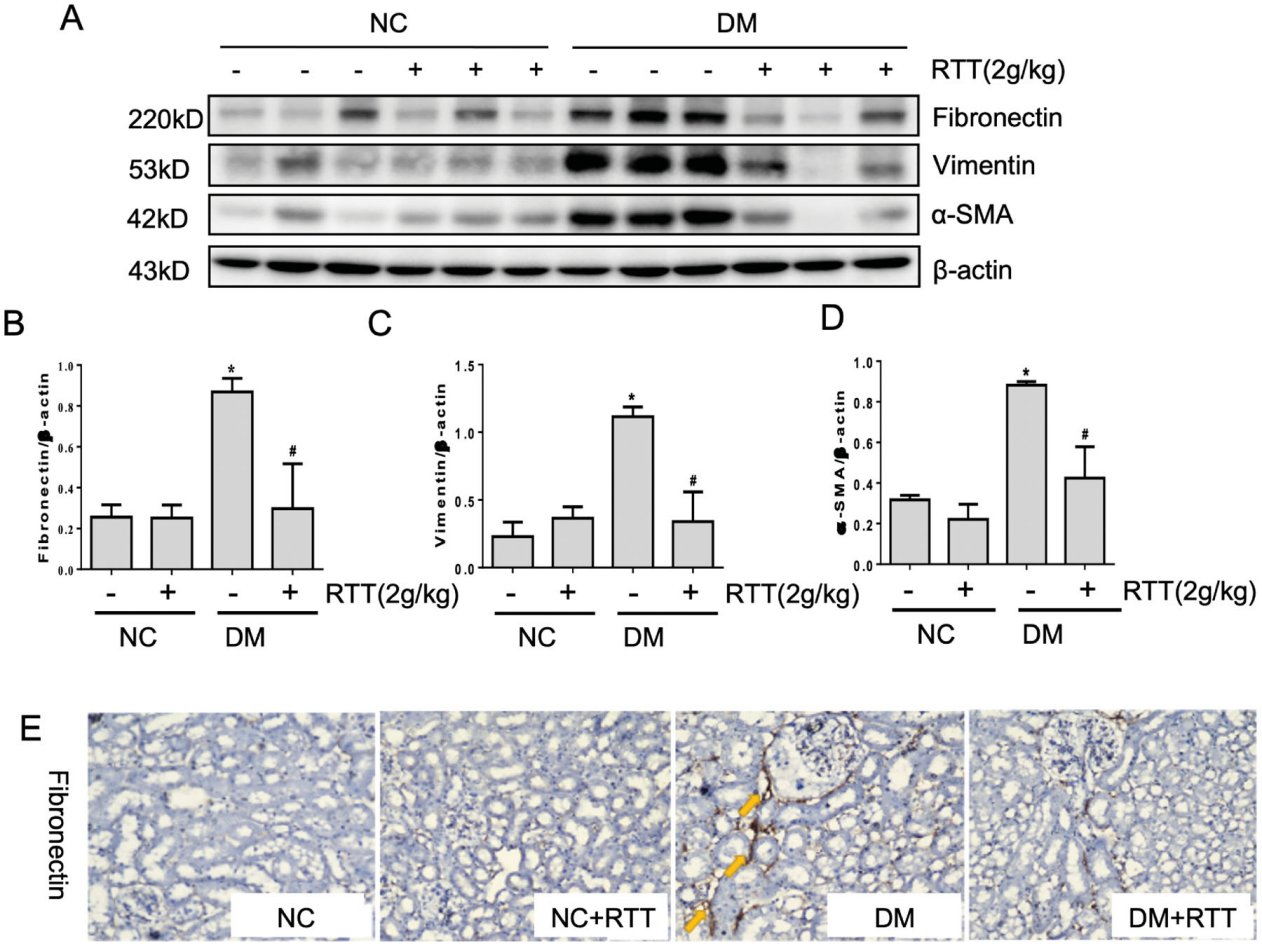
RTT extract inhibited renal fibrosis in STZ-induced diabetic mice. (A) Kidney tissue lysates were subjected to western blot analysis with antibodies against fibronectin, vimentin or α-SMA in STZ-induced diabetic mice after 28 weeks of RTT extract (2 g/kg) treatment (*n* = 12); Protein expression levels of fibronectin (B); Vimentin (C) and α-SMA (D) were normalized with β-actin (*n* = 12, means ± SD, *p < 0.05 vs. NC group; #*p* < 0.05 vs. DM group); (E) The expression of Fibronectin was detected by immunohistochemistry (original magnification, 400×, brown, yellow arrow) in STZ-induced diabetic mice after 28 weeks of RTT extract (2 g/kg) treatment (*n* = 12).

The above results demonstrated that RTT extract could alleviate renal damage and fibrosis in diabetic mice. Therefore, the expression levels of fibrosis-related proteins in renal tissues were measured by western blot and immunohistochemistry (Figure 4). The results showed that the levels of fibrosis-related proteins such as fibronectin (*p* < 0.05), Vimentin (*p* < 0.05) and α-SMA (*p* < 0.05) in STZ induced diabetic mice increased significantly, which could be further inhibited by RTT extract treatment (Figure 4(A–E)).

### RTT extract inhibited NF-κB signalling in STZ-induced diabetic mice

**Figure 5.**
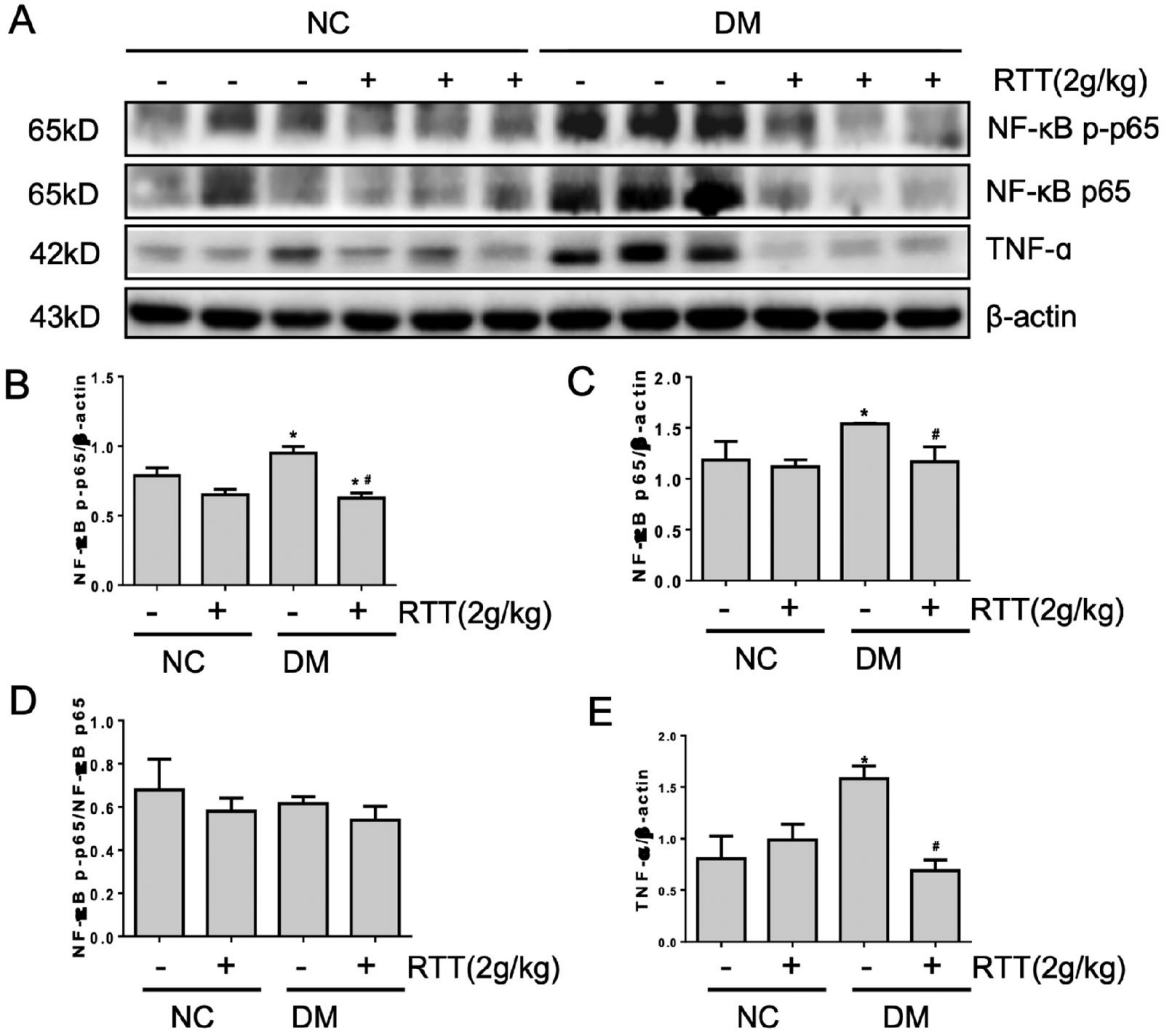
RTT extract inhibited NF-κB signalling in STZ-induced diabetic mice. (A) Kidney tissue lysates were subjected to Western Blot analysis with antibodies against NF-κB p-p65, NF-κB p65 or TNF-α in STZ-induced diabetic mice after 28 weeks of RTT extract (2 g/kg) treatment (*n* = 12); Protein expression levels of NF-κB p-p65 (B); NF-κB p65 (C); NF-κB p-p65/NF-κB p65 (D); TNF-α (E) were normalized with β-actin (*n* = 12, means ± SD, *p < 0.05 vs. NC group; #*p* < 0.05 vs. DM group).

Since inflammation is associated with DN (Zheng and Zheng 2016), this study explored whether RTT extract treatment can significantly alleviate the production of multiple inflammatory factors and related inflammatory signals in DN (Figure 5). Compared with normal mice, the expression level of NF-κB p-p65 (*p* < 0.05), NF-κB p65 (*p* < 0.05) and TNF-α (*p* < 0.05) increased in STZ-induced diabetic mice, and could be significantly alleviated by RTT extract treatment (Figure 5(A–D)).

### RTT extract inhibited SMYD2 in STZ-induced diabetic mice

**Figure 6.**
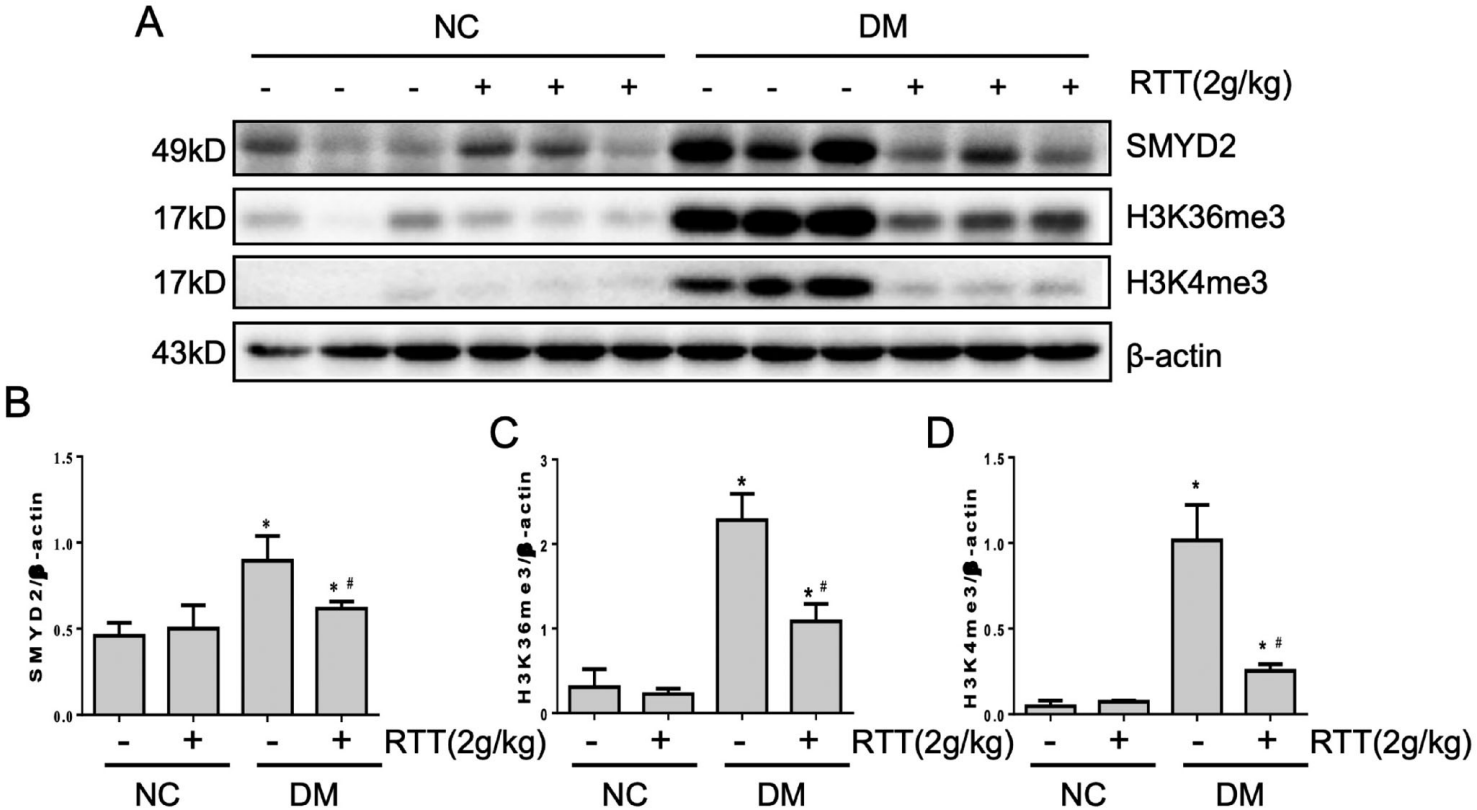
RTT extract inhibited SMYD2 in STZ-induced diabetic mice. (A) Kidney tissue lysates were subjected to western blot analysis with antibodies against SMYD2, H3K36me3 or H3K4me3 in STZ-induced diabetic mice after 28 weeks of RTT extract (2 g/kg) treatment (*n* = 12); Protein expression levels of SMYD2 (B); H3K36me3(C); H3K4me3 (D) were normalized with β-actin (*n* = 12, means ± SD, *p < 0.05 vs. NC group; #*p* < 0.05 vs. DM group).

It has been reported that activation of the NF-κB signalling pathway can be regulated by LMTs (Li et al. 2018). In this study, we explored whether RTT extract treatment could affect the expression level of SMYD2, H3K36me3 and H3K4me3 (Figure 6). In contrast to normal mice, STZ-induced diabetic mice expressed higher levels of SMYD2 (*p* < 0.05), H3K36me3 (*p* < 0.05) and H3K4me3 (*p* < 0.05), while RTT extract treatment dramatically reduced these levels (Figure 6(A–D)).

## Discussion

DN is the most serious complication of diabetes, which can be manifested as tubulointerstitial fibrosis and renal impairment (Li et al. 2021). Controlling blood glucose or treating the renal injury with currently available drugs have a variety of side effects (Kikiowo et al. 2020; Lartey et al. 2021). Therefore, it is very important to find alternative therapies based on Chinese herbal medicine with fewer side effects and multiple targets. RTT was first published in the Handbook of Chinese Medicinal Materials. Studies have shown that RTT has pharmacological effects such as clearing heat and detoxification, detumescence and dispersing nodules, relieving cough and resolving phlegm (Wu et al. 2015). Currently, it is mainly used in the treatment of tuberculosis and lymph node tuberculosis, as well as adjuvant therapy for cancer (Deng et al. 2013; Sun et al. 2013). Nevertheless, the role and mechanism of RTT in DN remain to be elucidated. Therefore, we investigated the anti-DN effects of RTT extract in STZ-induced diabetic mice.

In this study, the C57BL/6 diabetic mice were induced by STZ (55 mg/kg, five consecutive days), which develop similar renal injury to human DN (Sun et al. 2013). RTT extract (2 g/kg) was administered by gavage, and blood glucose levels were measured and analyzed. To explore the effect and molecular mechanism of RTT extract on renal fibrosis in DN, pathological changes and related protein expression changes were detected in the kidney. The results showed that the treatment of RTT extract did not affect blood glucose in diabetic mice, and HE and Masson staining showed a significant decrease in renal injury and fibrosis. The above results suggest that RTT extract has pharmacological effects on reducing renal injury and fibrosis.

In recent years, studies have shown that renal fibrosis is the main pathological feature of DN, manifested by renal interstitial inflammatory cell infiltration, fibroblast proliferation and activation, and ECM deposition, which ultimately leads to renal parenchymal destruction and renal failure (Chon et al. 2019). Among them, the excessive deposition of ECM is the main factor leading to renal interstitial fibrosis, and myofibroblasts are effector cells that synthesize and secrete ECM in this link. Under normal conditions, there are few myofibroblasts in the renal stroma; while under pathological conditions, fibroblasts proliferate, activate and transdifferentiate into myofibroblasts *in vivo*, which trigger the synthesis of ECM and renal fibrosis (Yuan et al. 2019). FN, Vimentin, and α-SMA are involved in the regulation of renal fibrosis (Choi et al. 2020; Tang et al. 2020) and are clinically important indicators for predicting the prognosis and efficacy of multiple chronic progressive nephropathies. Therefore, we investigated whether RTT extract reduced the expression of these molecules. The results showed that the treatment of RTT extract could inhibit the expression of several fibrosis markers in renal tissue of diabetic mice and show their anti-fibrosis properties. This study mainly focuses on the pharmacological effects of RTT, but the specific active ingredients of RTT also need to be further elucidated. Further study on the active components and specific action mechanisms of RTT will become the focus of future research. This study provides new ideas for developing drugs to prevent DN. And it is of great significance to further develop the medicinal value and clinical application of RTT.

SMYD2, one of the SET and MYND domain-histone methyltransferase, has been implicated in the development of some solid tumours (such as prostate and breast cancer) (Ding et al. 2018) and polycystic kidney, but its role in renal fibrogenesis has not yet been examined. In our previous research, we demonstrated that SMYD2 was highly expressed in STZ-induced renal fibrosis in DN. RTT extract treatment alleviates renal fibrosis. Therefore, inhibition of SMYD2 with RTT extract would be a promising therapeutic strategy for the treatment of renal fibrosis.

**Figure 7.**
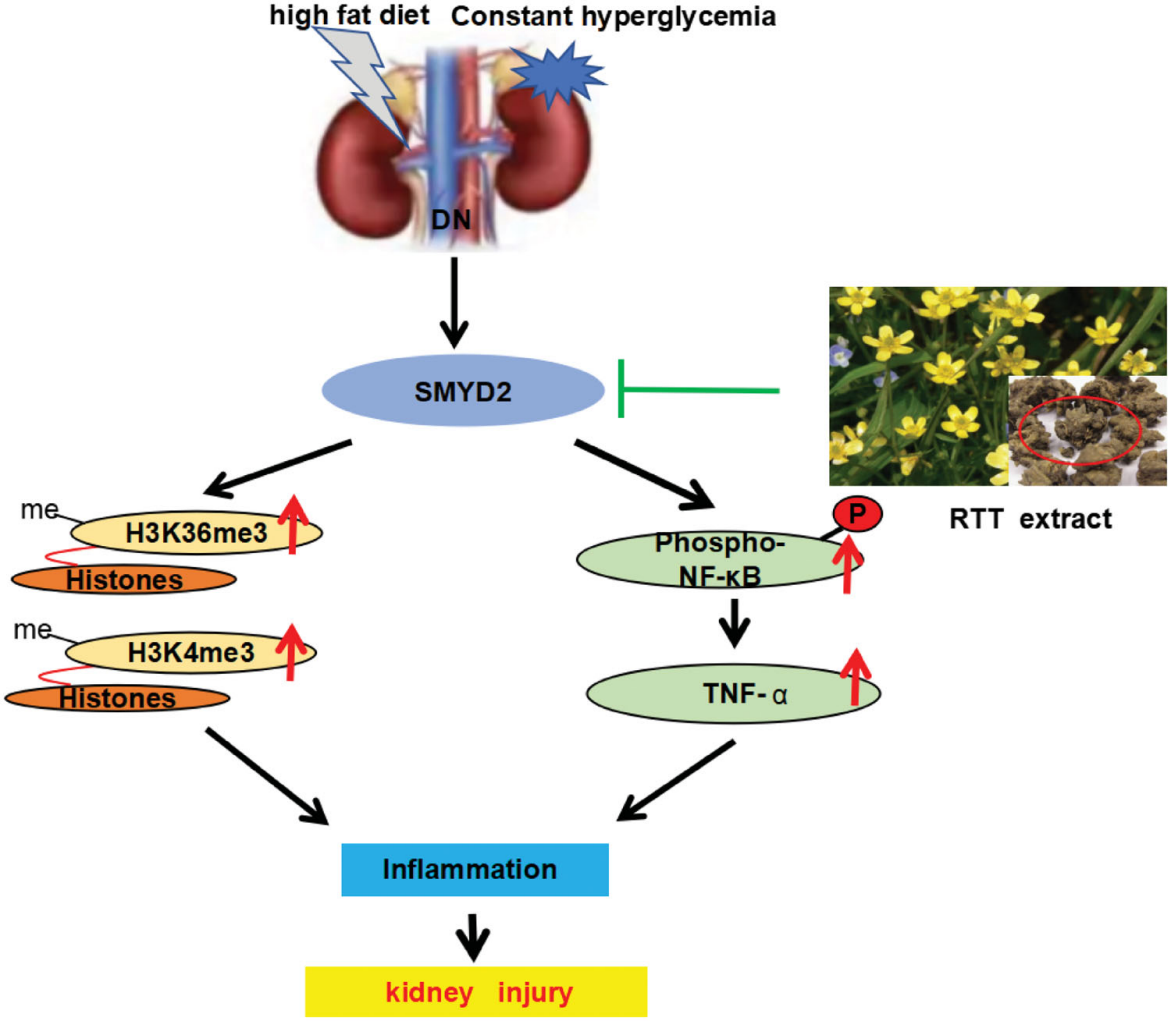
Proposed mechanisms of RTT extract inhibition SMYD2 mediated fibrotic effects in the kidney.

Studies have shown that SMYD2 was first identified as an H3K36-specific methyltransferase (H3K36me3) (Singh 2019). However, further studies have confirmed that SMYD2 can methylate H3K4 (H3K4ME3) or even non-histone (Abu-Farha et al. 2008; Yi et al. 2019), such as p53, STAT3 and NF-κB. NF-κB acts as a transcription factor that can be activated to induce the expression of chemokines and pro-inflammatory cytokines, leading to the recruitment and activation of immune cells. Activated immune cells in turn produce more pro-inflammatory cytokines/chemokines and growth factors, such as IL-1, IL-6, and TNF-α, which is closely related to the occurrence of DN development is closely related (Navarro-Gonzalez et al. 2011). In addition, TNF-α can also be further activated NF-κB by autocrine and/or paracrine. NF-κB plays an important role in the occurrence and development of DN by regulating the transcription of a variety of cytokines, promoting inflammatory infiltration and renal fibrosis (Li et al. 2020). Therefore, we speculated that the up-regulation of SMYD2 expression can activate various inflammatory factors and trigger the pro-fibrotic response (Figure 7).

To test this hypothesis, the expression of the molecules above was detected after RTT extract intervention. In STZ-induced diabetic mice, SMYD2, H3K36me3 and H3K4me3 were all highly expressed and accompanied by the activation of various inflammatory factors. Whereas RTT extract treatment remarkably reduced expression of SMYD2, NF-κB and TNF-α. The results demonstrate that SMYD2 is a key driver of renal fibrosis, and RTT extract alleviates renal fibrosis by regulating immune regulation and inhibiting SMYD2 (Figure 7).

## Conclusions

Our study demonstrated that RTT extract could significantly alleviate renal injury and fibrosis in STZ induced diabetic mice by regulating immune response, which is closely related to down-regulation and inhibition of SMYD2 protein expression, suggesting that SMYD2 plays a key role in regulating the occurrence and development of renal fibrosis.
